# Inactivation of Dermatophytes Causing Onychomycosis Using Non-Thermal Plasma as a Prerequisite for Therapy

**DOI:** 10.3390/jof7090715

**Published:** 2021-08-31

**Authors:** Eliška Lokajová, Jaroslav Julák, Josef Khun, Hana Soušková, Radim Dobiáš, Jaroslav Lux, Vladimír Scholtz

**Affiliations:** 1 Department of Physics and Measurements, Faculty of Chemical Engineering, University of Chemistry and Technology Prague, Technická 5, 166 28 Praha, Czech Republic; khunj@vscht.cz (J.K.); vladimir.scholtz@vscht.cz (V.S.); 2 Institute of Immunology and Microbiology, First Faculty of Medicine, Charles University, Studničkova 7, 128 00 Praha, Czech Republic; jaroslav.julak@lf1.cuni.cz; 3 Department of Computing and Control Engineering, Faculty of Chemical Engineering, University of Chemistry and Technology Prague, Technická 5, 166 28 Praha, Czech Republic; Hana.Souskova@vscht.cz; 4 Department of Bacteriology and Mycology, Public Health Institute in Ostrava, Partyzánské Nám. 7, 702 00 Ostrava, Czech Republic; radim.dobias@seznam.cz; 5 Department of Biomedical Sciences, Institute of Microbiology and Immunology, Faculty of Medicine, University of Ostrava, Syllabova 19, 703 00 Ostrava, Czech Republic; 6 Podiatric Center Medicia, Daliborova 421/15, 709 00 Ostrava, Czech Republic; jaroslav.lux@seznam.cz

**Keywords:** DC corona discharge, *Microsporum*, treatment of mycoses, *Trichophyton*

## Abstract

Following our previous study of the therapy of onychomycosis by non-thermal plasma (NTP) and nail hygiene and to obtain some prerequisite data of dermatophytes sensitivity, the dynamics of those inactivation by NTP plasma was monitored for various strains of *Trichophyton iterdigitale*, *Trichophyton benhamiae*, *Trichophyton rubrum*, and *Microsporum canis*. Three strains of each species on agar plates were exposed with plasma produced by a DC corona discharge in the point-to-ring arrangement in various time intervals. Although all strains were sufficiently sensitive to plasma action, significant differences were observed in their sensitivity and inactivation dynamics. These differences did not correlate with the species classification of individual strains, but could be assigned to four arbitrarily created types of strain response to NTP according to their sensitivity. These results indicate that the sensitivity to plasma is not an inherent property of the fungal species, but varies from strain to strain.

## 1. Introduction

Micromycetes, filamentous microscopic fungi or molds, can cause food degradation. They are important in medicine as causes of general infections (invasive mycoses), mycotoxicoses and allergies, as well as local infections (superficial and mucosal mycoses, dermatomycoses). This group of dermatophytes includes mainly the genera *Trichophyton* and *Microsporum*, containing approximately ten species of parasitic fungi. These species are less dangerous, but they are important causative agents of benign, but annoying superficial mycoses in humans referred to as *tinea corporis*, *tinea barbae*, *tinea capitis*, onychomycosis, etc. For therapy, topically applied preparations based on imidazoles, allylamines, pyridines, or morpholines are recommended. Nevertheless, the relatively low efficacy of the classical therapy has motivated efforts to apply new physical therapy methods.

Recently, non-thermal plasma, (NTP, also called low-temperature plasma) has been reported as a tool for fungal inactivation. Plasma, also called the fourth state of matter, is a partially or fully ionized gas. There is a distinction between high-temperature plasma, reaching temperatures of thousands of kelvin, and NTP, which occurs at nearly ambient temperature and contains low-temperature ions and highly energetic free electrons. NTP is a partially ionized gas where most of the energy is stored in electrons, whereas other particles remain at room temperature. NTP may be easily obtained by various electric discharges between point or plane electrodes at high voltage. Free electrons are accelerated by an electric field and generate secondary electrons, free radicals, ions, and photons by collisions with neutral particles. The most commonly used discharges are corona discharges, plasma jets (also called plasma needle, plasma torch, or plasma pen), dielectric barrier discharge, gliding arc, and microwave discharges. For a more detailed description of plasma sources, see papers [1,2,3]. A special DC discharge called cometary was described by Scholtz and Julák [4,5] and used, among others, by Wong et al. [6]. The point-to-ring arrangement of the corona discharge has been applied in onychomycosis therapy [7]. The basic knowledge about NTP can be found in a review article by Moisan et al. [8].

The ionized gas produced by NTP contains a cold mixture of free radicals and charged particles, which does not substantially increase the temperature of the target object. The microbicidal activity of NTP is mediated mainly by reactive oxygen particles (ROS) and reactive nitrogen particles (RNS) arising from the surrounding gases. Various species such as ions, radicals, and stable or unstable electroneutral molecules, namely, superoxide anion, singlet oxygen, hydroxyl and hydroperoxyl radical, nitric oxide radical, peroxynitrite, and others, may be present. The lifetimes of these species are very short, but stable compounds are also formed, namely, hydrogen peroxide, ozone, and nitrogen oxides [9,10,11,12]. Although Cheng et al. [13] presented an overview of ROS action mechanisms, the detailed mechanisms of the biological effects of NTP on unicellular microbes are still poorly understood; apart from physical destruction and necrosis, apoptosis also occurs in unicellular microbes including yeasts. As was described, the exposure of bacteria or yeasts to NTP not only induces direct physical destruction, but also triggers programmed cell death [14]. Some hallmarks of apoptosis were also found in *Pseudomonas aeruginosa* [15] and in yeasts [16].

The mechanisms of fungal spores inactivation by ROS produced by the non-equilibrium atmospheric-pressure plasma were carefully studied by Ito et al. [17]. Inter alia, Ali et al. [18] or Borges et al. [19] have described the in vitro inactivation of *Trichophyton* spp. Bulson et al. [20] described the same effects applied on dermatomycosis treatment on in vitro human nail model. Among in vivo applications, the superficial mycosis treatment [21] and onychomycosis treatment may be mentioned. In it [7] was shown that, under certain conditions, plasma applications are also effective in human therapy the treatment of patients with onychomycosis.

In general, different microbes exhibited different sensitivity to NTP; while bacteria could be completely inactivated within seconds to minutes, yeasts required exposure for several minutes and mold spores can survive exposures lasting several tens of minutes. Comparable exposure times are required to deactivate sporulated bacteria [5] or microorganisms in the form of a biofilm, which are considerably more resistant to the microbicidal action of plasma in comparison with their planktonic forms [22]. Different species of fungi showed significant differences in the time of exposure needed to inactivate them. For example, *Cladosporium sphaerospermum* spores were completely inactivated within 10 min, whereas *Aspergillus oryzae* spores were not inactivated even after 40 min under the same conditions; *Alternaria* spp. and *Byssochlamys nivea* exhibited intermediate sensitivity [23]. Paper [24] presented similar results: Whereas total inactivation of yeast occurred in six minutes, spores needed 20–25 min of exposure in the case of *Cladosporium sphaerospermum* and *Penicillium crustosum*; *Aspergillus oryzae* spores were not completely inactivated even after 30 min of exposure. Scholtz et al. [25] described the sensitivity of dermatophytes: The anthropophilic and zoophilic species *Trichophyton rubrum* and *T. interdigitale* were found to be highly sensitive to NTP both in suspension and on surfaces, and so was zoophilic *Arthroderma benhamiae*. In contrast, the geophilic species *Nannizzia gypsea* appeared as highly resistant. In addition, significant differences were observed in all of these studies between various modes of NTP production, namely, between positive and negative DC corona or between corona and dielectric barrier discharge [23].

In this study, we tried to contribute to the explanation of the mechanisms of plasma action on micromycetes by describing the dynamics of this action. We follow our previous study described in Lux et al. [7] where we demonstrate the therapeutic possibilities of NTP on three dermatophytes species in a wide range of multiple exposure schemas. Here, we would like to focus on mutual comparisons of four dermatophytes species, each in three strains, to determine the trends of their sensitivity to NTP treatment resulting in some therapeutic recommendations. The successful therapeutic effect was observed in some cases in combination with nail hygiene, so the aim of this work is to determine whether susceptibility to NTP is a characteristic of individual fungal species. Indeed, our previous results have shown that differences in plasma efficacy may depend on the growth phase of the fungus in which it was applied. Therefore, we have also attempted to contribute to the elucidation of the mechanism of action of the plasma by describing the dynamics of this action. Specifically, we have created a more detailed analysis of the susceptibility to NTP for four species, each in three strains, of clinical isolates of most common dermatophytic micromycetes to find out the trends of their sensitivity to NTP treatment, resulting in some therapeutic recommendations.

## 2. Materials and Methods

### 2.1. Plasma Source

**Figure 1:**
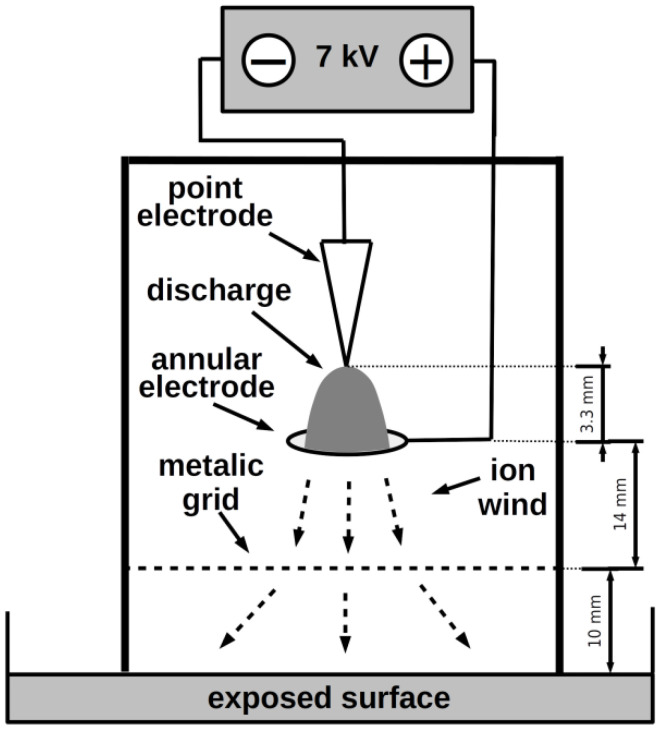
Schematic arrangement of the point-to-ring discharge.

NTP was produced by a negative DC corona discharge in the point-to-ring arrangement, depicted schematically in Figure 1. The working electrode was made from a medical injection needle; the other annular electrode of 12 mm in diameter was made of brass and was approx. 3.3 mm below the tip of the needle electrode. The electrodes were connected to the constant DC voltage source of 7 kV and the current was adjusted to 150 µA. The taken spectrum of the discharge was very similar to that of negative point-to-plane corona or pulseless glow discharge as presented in our previous study [2], so the discharge was considered to be the normal mode of negative corona. An embedded metallic grid on floating potential was used to disperse generated particles homogeneously to the whole area of exposed surface; the surface of exposed agar medium in Petri dish was electrically isolated. This arrangement prevents the uneven strongest action of the plasma in the middle of the dish and its drop towards the edges; for details, see Julák et al. and Scholtz et al. [26,27].

### 2.2. Micromycete Strains

The following strains of dermatophyte micromycetes were used: *Trichophyton iterdigitale* strains 5937-2017, 7371-2017, and 7503-2014; *Trichophyton benhamiae* strains 5051-2014 and 6590-2017; *Trichophyton rubrum* strains 2360-2019, 6092-2018, and 9250-2018; *Microsporum canis* strains 1714-2018, 8446-2017, and 8564-2017. The selected strains were already used in a previous our study [7]. The experimental fungal strains used in the study were chosen according to the current epidemiological situation in the Czech Republic where *T. rubrum* and *T. interdigitale* are the most common anthropophilic and zoophilic agents of onychomycosis, respectively. Isolates of *T. benhamiae* were added to this experiment as a marginal zoophilic etiological agent of onychomycosis and with *M. canis* as dominant etiological agent of tinea corporis. [28,29]. All strains were obtained from the collection of the Public Health Institute in Ostrava, Czech Republic. Isolates of study strains were grown on a set of agar media including malt extract agar agar (MEA; Oxoid, Basingstoke, UK), Sabouraud dextrose agar (SDA; HiMedia, Mumbai, India), and potato dextrose agar (PDA; HiMedia) at 25 and 37 °C. Species identification of all dermatophytes was determined by micro- and macro-morphology characteristics and confirmed by internal transcribed spacer (ITS) region of rDNA sequencing (ITS1-5.8S-ITS2 cluster). All strains were pre-cultivated on Sabouraud agar at 25 °C until signs of culture sporulation appeared after 4–10 days, depending on the strain. From sporulating primocultures, the stock suspensions were prepared in 4 mL of water with 25 μL of Tween 80 added to improve the wettability of the spores. The concentration of stock suspension was adjusted to 1000–2000 cfu/mL (colony forming units per mL). The experimental Sabouraud agar was inoculated with 100 μL of stock suspensions according to the scheme described in the following paragraph. Thus, the final concentration of micromycetes was 100−200 cfu per agar plate.

### 2.3. Exposure Arrangement

**Figure 2:**
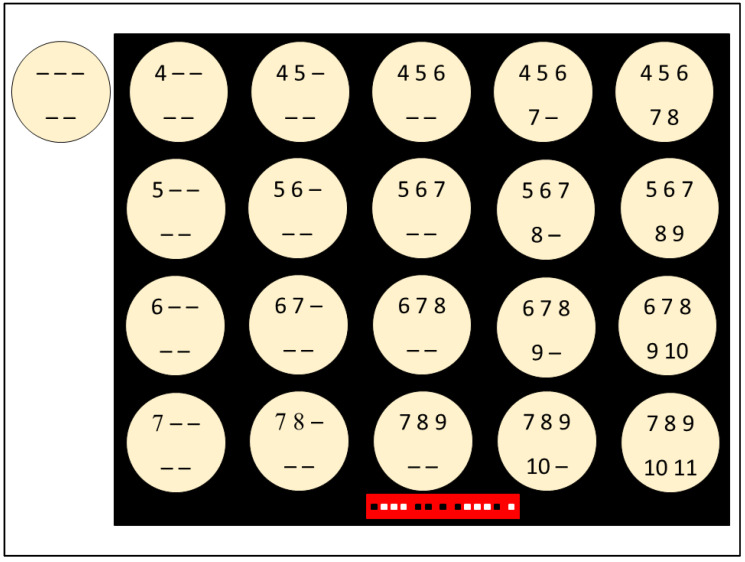
Schematic timing of exposure of experimental dishes. Numbers indicate exposures in days. Multiple digits equal multiple exposures.

Each strain suspension was inoculated onto the whole surface of one unexposed control plate and onto 20 Sabouraud agar experimental plates which were subsequently exposed to plasma, as described below. The inoculated plates were incubated at 22–25 °C for 20 days, during which they were consecutively exposed to NTP according to the scheme shown on Figure 2. The numbers in the circles (plates) indicate the day(s) when the dish was exposed, i.e., number 4 indicates exposure on the fourth day after inoculation, 4 and 5 on the fourth and fifth day after inoculation, etc. A dash (-) means that the dish was not exposed on that day; therefore, the unexposed control dish contains only dashes. The intervals between exposures were 24 h; the duration of each individual exposure was 10 min.

### 2.4. Evaluation

**Figure 3:**
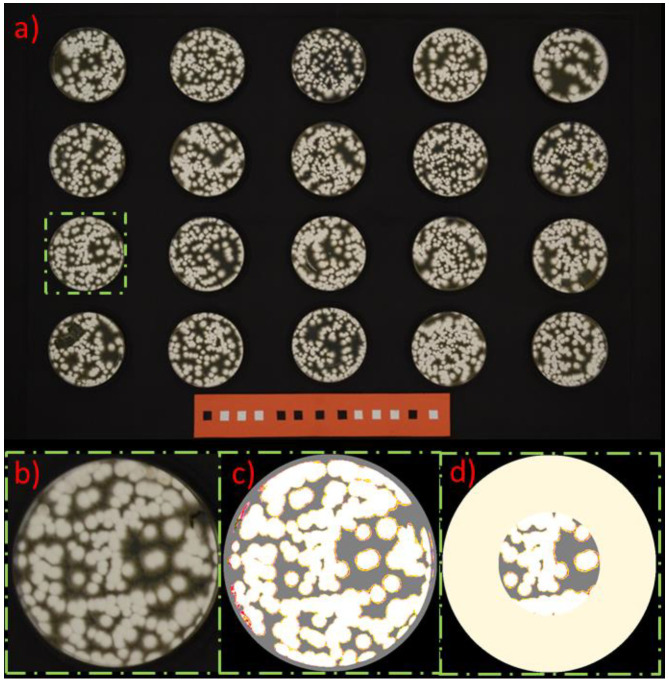
Example of the plate evaluation: (**a**) original plate; (**b**) cut of sample; (**c**) threshold for distinguishing inhibited area; (**d**) cut of the exposed area.

Each set of plates containing cultures of one individual strain was photographed daily in a lighting box with a NIKON D3100 camera. The images were processed by image analysis. For the image analysis, single purpose self-made program for automatic evaluation of different color area to distinguish between grown and clean area was used. To evaluate the growth of micromycetes on the dish, the percentage of the area on the exposed part of the dish overgrown with micromycete was chosen as a criterion. The white area of the fungi on the yellow-brown surface of ungrown agar allowed the delineation of the overgrown area. One illustrative example is shown in the Figure 3.

The determined values of the areas covered on individual dishes with the culture of the fungus concerned, measured for individual exposures, were recorded in the tables. However, the complete tables are very voluminous for publication in extenso and therefore they are presented in a separate appendix under Supplementary tables only. The condensed trends of the groups in which the monitored strains could have been included according to the general nature of their response to NTP are presented in the Results section. The model of micromycetes growth in time is represented as the overgrown area *G(t)* based on second order system with transport delay [30,31] resulting in the formula:

(1) $$G\left(t\right)=\theta \left(t-∆t_{0}\right)\cdot \left(\alpha \cdot e^{-a\cdot t}-\beta \cdot e^{-b\cdot t}+\varphi \right),$$

where θ is the Heaviside step function; Δ*t*_0_ represents the time delay between inoculation and the first visible growth; *α*, *β*, *a*, *b* are parameters of shape of the function; and the parameter *φ* determines the final value of *G* extrapolated to infinite time *t*→∞.

## 3. Results

**Figure 4:**
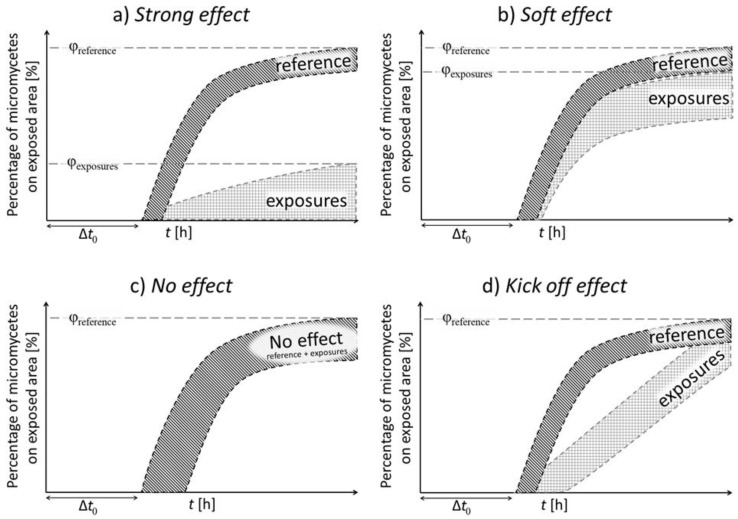
The characteristic growth types of exposed micromycete strains after NTP exposure. (**a**) Strong effect type; (**b**) Soft effect type; (**c**) No effect type; (**d**) Kick off effect type.

The studied strains can be divided into four types according to their growth trends observed after plasma exposure. Figure 4 shows the characteristics of these types. The particular types are defined as follows: The “Strong effect” type (a) displayed almost complete growth suppression after exposure. In contrast, the “No effect” type (c) included strains whose growth was not inhibited at all. The intermediate type “Soft effect” (b) included strains whose growth was only slightly inhibited. In the “Kick off effect” type (d), an initial slowdown of growth, followed by a rapid growth, was observed.

**Figure 5:**
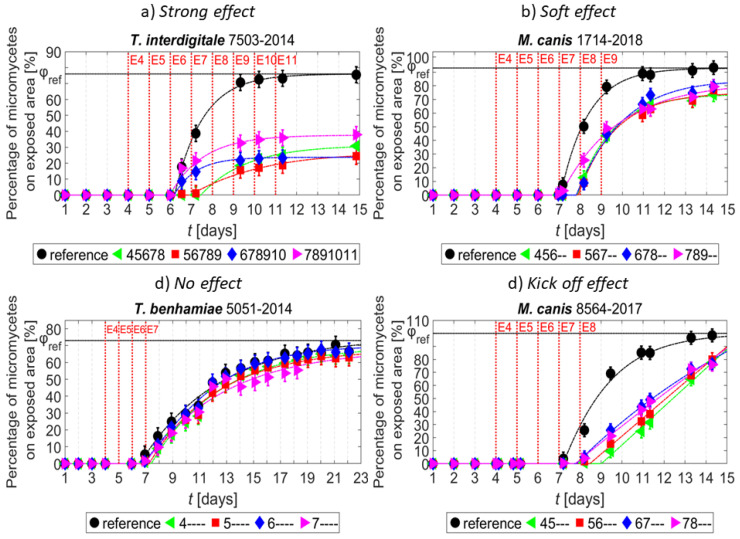
Selected examples of growth curves for each type of growth. (**a**) Strong effect type; (**b**) Soft effect type; (**c**) No effect type; (**d**) Kick off effect type.

Some individual strains selected as typical examples of the categories defined above are shown in Figure 5. In Figure 5a, the strong inhibition of growth of exposed samples is apparent the shape of the curves is similar to the reference, but the final overgrowth is less than one half of the reference. Figure 5b shows the “Soft effect” type similar to the “Strong effect” type, but the final values are higher than one half of reference. Figure 5c demonstrates the “No effect” type, where all curves are similar to the reference one. Finally, Figure 5d shows the “Kick off effect” type, where the shape of the exposed samples curves is linear; the beginning of growth is delayed in time, followed by higher growth speed, reaching almost the same values as the reference.

**Figure 6:**
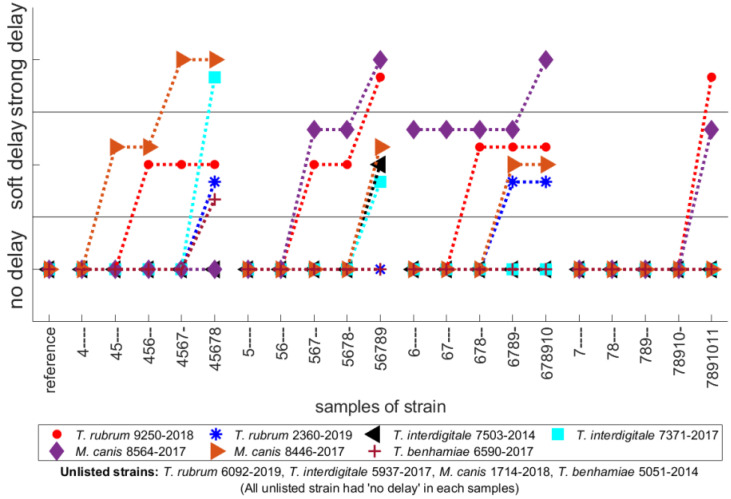
The time delays for particular strains and all exposures; no delay occurred for unlisted strains.

Concerning the influence of NTP exposure on the time delay Δt_0_, the strains may be characterized and divided into three groups: (a) Strong delay for delay higher than four days after reference; (b) soft delay for delay between two and four days; (c) no delay for delay lower than two days. The overall dependence of the delay for individual samples are shown in Figure 6. NTP is able to delay the visible growth for several strains only and it depends on the day of first exposure, and also on the total number of exposures. For *M. canis* 8446-2017 and *T. interdigitale* 7371-2017, the higher delay occurred for multiple exposures beginning at 4th day. However, for *M. canis* 8564-2017 there is strong delay on the 4th day, but a no delay on the 5th, 6th, and 7th day with the same multiple exposures.

**Figure 7:**
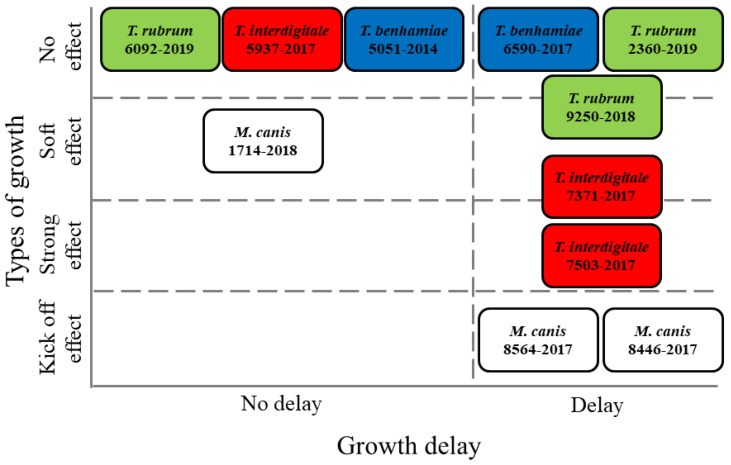
Classification of particular micromycete strains with respect to growth trends.

The assignment of the individual strains observed in this study to the types defined above is summarized in Figure 7.

## 4. Discussion

Designing this work, we assumed that the fungal species should be characterized by its sensitivity to plasma exposure. However, it appeared that this is an individual property of various strains of the same species, which are heterogeneous with respect to this feature. It is obvious that the inclusion of strains into the above-described type schemes does not correspond to the taxonomic classification of the monitored species i.e., that strains classified as the same species fall into different categories of classification according to plasma sensitivity. Per analogiam, a similar phenomenon occurs frequently among bacteria, where virulent and non-virulent strains of the same species are common. *Escherichia coli* may serve as a typical example: this species is mostly commensal but contains also pathogenic (virulent) subspecies (strains of various serovars). This special marker is not considered in bacterial taxonomy but is of crucial importance in human pathogenesis. Similar examples of the sensitivity to plasma was reported in our previous study [15], where the collection strain and the clinical isolate of *Pseudomonas aeruginosa* differ markedly. We do not propose that discrepancies in a single marker, which may be provisionally labeled as “Plasma Sensitivity”, should be suitable in the taxonomy of the micromycetes concerned. After all, the observed strains showed significant differences in growth rate even without plasma exposure. At most, it is possible to consider the classification of the studied strains into subspecies differing only in this feature. All strains of dermatophytes have been obtained from clinical material and it may be clones of different strains of one species that have occurred in different types of environments (“same species are not the same”). This may affect the expression of genes related to cell wall structures. During host adaptation, the cell wall structure of fungal pathogens is continuously reshaped by the orchestrated action of numerous genes [32]. The changes in the ambient environment caused continuous cell wall remodeling, forcing the fungus to undergo modulatory restructuring and this fact could affect the sensitivity to the stable compounds arising during NTP application, such as hydrogen peroxide, nitrogen acids and probably also ozone. [23,33,34,35] Clarification of these mechanisms should be part of future research. The solution to this intra-species inherence in the sense of the NTP susceptibility could be longer exposure of dermatophytes to NTP. From a practical point of view, these findings suggest that, contrary to our expectations, the sensitivity of any clinical findings of these micromycetes to the therapeutic effect of applied plasma therapy cannot be estimated. This leads to a rather unpleasant conclusion that the identification of a fungal species causing a human disease may not predict the parameters of a possible plasma therapy or its success. We have observed this phenomenon in our previous attempts in the treatment of onychomycosis [7]. On the other hand, this study showed an optimistic conclusion that NTP may be useful in the treatment of onychomycosis, but only under certain conditions. A necessary condition for efficacy is probably that complete inactivation of the fungus can only be achieved when plasma is applied at an early stage of growth. A practical implication of this finding for therapeutic applications is the necessity of preliminary nail plate abrasion and refreshing prior to NTP application.

## Data Availability

The data presented in this study are available on request from the corresponding author.
